# Multi-criteria decision analysis (MCDA): testing a proposed MCDA framework for orphan drugs

**DOI:** 10.1186/s13023-016-0555-3

**Published:** 2017-01-17

**Authors:** C. Schey, P. F. M. Krabbe, M. J. Postma, M. P. Connolly

**Affiliations:** 1 Unit of PharmacoEpidemiology & PharmacoEconomics, Department of Pharmacy, University of Groningen, Groningen, The Netherlands; 2 Global Market Access Solutions (GMAS), St-Prex, Switzerland; 3 Department of Epidemiology, (UMCG), University of Groningen, University Medical Center Groningen, Groningen, The Netherlands; 4 Institute for Science in Healthy Aging & healthcaRE (SHARE), UMCg, University of Groningen, Groningen, The Netherlands

**Keywords:** Multi-criteria decision analysis, MCDA, Orphan drugs, Reimbursement, Mucopolysaccharidosis II, Paroxysmal nocturnal haemoglobinuria, Pulmonary arterial hypertension, Myelodysplastic syndromes, Lennox-Gastaut syndrome

## Abstract

**Background:**

Since the introduction of the orphan drugs legislation in Europe, it has been suggested that the general method of assessing drugs for reimbursement is not necessarily suitable for orphan drugs. The National Institute for Health and Clinical Excellence indicated that several criteria other than cost and efficacy could be considered in reimbursement decisions for orphan drugs. This study sought to explore the multi-criteria decision analysis (MCDA) framework proposed by (Orphanet J Rare Dis 7:74, 2012) to a range of orphan drugs, with a view to comparing the aggregate scores to the average annual cost per patient for each product, and thus establishing the merit of MCDA as a tool for assessing the value of orphan drugs in relation to their pricings.

**Methods:**

An MCDA framework was developed using the nine criteria proposed by (Orphanet J Rare Dis 7:74, 2012) for the evaluation of orphan drugs, using the suggested numerical scoring system on a scale of 1 to 3 for each criterion. Correlations between the average annual cost of the drugs and aggregate MCDA scores were tested and plotted graphically. Different weightings for each of the attributes were also tested. A further analysis was conducted to test the impact of including the drug cost as an attribute in the aggregate index scores.

**Results:**

In the drugs studied, the *R* ^2^, that statistically measures how close the data are to the fitted regression line was 0.79 suggesting a strong correlation between the drug scores and the average annual cost per patient.

**Conclusion:**

Despite several limitations of the proposed model, this quantitative study provided insight into using MCDA and its relationship to the average annual costs of the products.

## Background

In recent years, substantial criticism has arisen regarding the way in which orphan drugs are reviewed in health technology assessments (HTAs), in particular with respect of their inability to meet cost-effectiveness standards for reimbursement decisions [1]. In some countries, the orphan drugs are reimbursed despite their lack of meeting cost-effectiveness thresholds, yet in others, such as in Scotland, reimbursement has been denied on the basis of a lack of cost-effectiveness. For example, alglucosidase alfa was refused reimbursement on the basis that “*The economic case has not been demonstrated”* [2]. Information regarding the cost-effectiveness of orphan drugs is derived from economic evaluations, such as cost-effectiveness analyses or cost-utility studies [3]. Such studies involve comparing the new drug with the existing treatment options [4]. The perceived advantage﻿ of the current HTAs – that includes cost-effectiveness - is that they provide an output, the incremental cost-effectiveness ratio (ICER) that is intended to make the outcomes of the different technologies comparable across different diseases. However, there is concern that valuing healthcare interventions mostly based on cost-effectiveness is a form of healthcare rationing, in particular since cost-effectiveness only takes two criteria (cost and efficacy) into consideration, and that using the ICER may limit the potential treatment options available to patients by excluding potentially worthwhile alternatives [5]. For example, in England, the National Institute for Health and Care Excellence (NICE) has adopted a nominal cost-per-QALY threshold of £20,000 to £30,000 [6]. However, if the cost-effectiveness analysis of a drug yields a cost-per-QALY that is substantially higher than the guide threshold, it might be refused funding.

Furthermore, concern has been raised that because of their costs, in addition to the frequent lack of suitable comparators, and the difficulties of demonstrating robust efficacy in small patient populations, orphan drugs are not deemed (robustly) cost-effective under the standard methods of HTA [1, 3]. This potentially results in patients not having access to potentially valuable treatments [7].

While the debate regarding the applicability of cost-effectiveness analysis for orphan drugs has been ongoing, multi-criteria decision analysis (MCDA) has been suggested as an alternative to the standard HTA methodology for assessing orphan drugs [8]. MCDA is a methodology for supporting decision making when multiple objectives, aside from cost and efficacy, have to be assessed [9]. For example, other aspects that may need to be considered are the availability of alternative treatments, disease prevalence and disease severity. MCDA has been extensively used to support a wide variety of complex decision problems in non-health industries, such as geographic information systems [10], banking and finance [11] and environmental policy issues for many years [12]. In the last few years, interest in the use of MCDAs in healthcare has increased. MCDA has been adopted in a number of studies in healthcare [13–15]. MCDA has not yet been used exclusively in HTAs in place of cost-effectiveness studies, although The National Institute for Health and Care Excellence (NICE) and the Scottish Medicines Consortium (SMC) in the UK occasionally adopt an MCDA approach, in that they consider cost-effectiveness as well as several other criteria [16, 17].

MCDAs provide a structured framework for the comparison of multiple options (or criteria) relating to a drug or a disease. Through engagement with a broad range of potential stakeholders, that can include clinicians, decision makers and the public, MCDAs allow the different perspectives for the preferences of the criteria and their relative importance in rare diseases to be taken into account [18, 19]. Because MCDAs offer a number of ways of aggregating the data of the individual criteria, they inherently provide a system for ranking healthcare interventions [20]. Through weighting the importance of the different criteria, MCDAs allow for clear trade-offs between various criteria [21, 22]. One of the main points, and indeed part of the impetus for change by health services, in particular the National Health Service (NHS) in the UK, is the aim of including wider aspects of social and economic value in healthcare assessments, rather than to use “health gain” as the sole currency of value in the NHS [23]. Because it is designed to consider a broad set of criteria and their values, the MCDA offers a framework that is robust, transparent and can be flexible in assessing orphan drugs for different diseases [24, 25], and in particular fills a current void in appropriate mechanisms to assess the value of orphan drugs.

Considering the criticisms of existing HTA processes, several attempts have been made at using alternative methods to inform decision makers on the appropriate allocation of healthcare funds. In 2012, Hughes-Wilson et al. [8] developed an MCDA algorithm that assesses a medicine based on multiple criteria. The aims of this study were to apply the MCDA framework that was proposed by Hughes-Wilson et al. [8] to a range of orphan drugs in different diseases, with a view to testing the relationship between drug price and aggregated MCDA scores for each product.

## Methods

### MCDA Framework

An MCDA framework was developed using the nine suggested criteria [8], which included: *Rarity, level of research undertaken, Level of uncertainty of effectiveness, Manufacturing complexity, Follow-up measures, Disease severity, Available treatment alternatives, Level of impact of disease, and Unique indication or not.* The *Follow-*up *measures* refers to any additional requirements by regulatory or similar authorities. The *Level of impact of disease* refers to the extent to which the new technology impacts on the disease in question. Each of these criteria are further described in Table 1.

**Table 1:** Description of criteria used in this study

Criteria	Category	Score
Rarity	Based on published prevalence data: 1:2,000–1:20,000	1
	1:20,000–1:200,000	2
	<1:200,000	3
Level of research undertaken	“Blue sky”	1
	Building on previous knowledge	2
	Literature review	3
Level of uncertainty of effectiveness	Immature but promising data	1
	Appropriate surrogate endpoints	2
	Robust clinical endpoints	3
Manufacturing complexity	Not complex; small molecule	1
	Moderately complex	2
	Highly complex, biological and galenic form	3
Follow-up measures	Safety and efficacy studies, and size and duration of study	1
	Designed to answer specific, defined delineated question	2
	Moderate to none	3
Disease severity	Morbidity	1
	Mortality, severe invalidity in adulthood	2
	Mortality/severe invalidity as an infant	3
Available alternatives/Unmet needs	Alternatives with similar characteristics	1
	Alternatives - but this offer strong innovation to disease treatment	2
	No alternative	3
Treatment impact on disease	Low	1
	Medium	2
	Strong	3
Unique indication or not	Existing orphan or non-orphan indication for the same molecule	1
	Potential for multiple indications	2
	Unique indication. No other possible use	3

Six orphan drugs were identified for the prototype evaluation for which data were obtained from Summary of Product Characteristics (SPC) and European public assessment reports (EPAR). A literature search was performed to identify relevant clinical trials for each of the drugs. Data relating to the results for the criteria included in the study were extracted. Furthermore, the literature review included disease-specific peer-reviewed publications and patient advocacy organisations to extract data relating to disease severity and the impact of disease on patients.

The six drugs were selected for the study based on the wide range of average annual cost per patient for each drug, as well as the wide range of diseases which they treat. Furthermore, some were included due to the consistently negative media coverage they receive. The average annual cost is based solely on the cost of the drugs, and therefore excludes any additional costs, such as those related to administration of the drugs or any ancillary items for intravenously administered drugs. These drugs were also selected to represent different degrees of the rarity of the diseases they treat, based on the categorisation provided in the proposed framework on which this study is founded. For example, a disease with a prevalence of 1 per 2,000 to 1 per 20,000 population (highest prevalence level) scored 1. The drugs included are indicated for: Pulmonary arterial hypertension (PAH), Mucopolysaccharidosis VI (MPS VI), Mucopolysaccharidosis II (MPS II), Paroxysmal nocturnal haemoglobinuria (PNH), Lennox-Gastaut syndrome (LGS) and Myelodysplastic syndromes (MDS).

Based on the scoring approach suggested [8], each criterion was allocated a numerical score, from 1 to 3, where 1 indicated the lowest level of attribution allocated. This simple numerical scoring system avoids the need for an expert panel to score the different criteria. The average dose per patient was calculated from product SPCs, taking account of the dose variation for drugs that are used in children (e.g. Mucopolysaccharidosis VI), versus those most likely to be used only in adults (e.g. Myelodysplastic syndromes) without any specific weighting of paediatric versus adult use. Using the average dose, the average annual cost per patient was calculated from published prices in the British National Formulary (BNF) and converted into Euros (January 2014 exchange rates). Due to pricing confidentiality, official list prices were adopted for the drugs included in this study. For the purposes of this model, all drugs were assumed to be used routinely as indicated in the SPC for a total of 1 year.

### Analysis

The relationship between the average annual cost of each drug and aggregate MCDA scores were tested and plotted graphically. Weighting preferences, as tabulated in Table 1, were applied to the different criteria to assess how the results might differ. The weighting preferences were not based on primary data collection but rather by using 3 scenario tests. In the first scenario, all criteria were weighted equally. In the second scenario, the criteria of *Level of Research Undertaken*, *Level of Effectiveness Uncertainty, Manufacturing Complexity and Unique Indication or Not* were excluded, on the basis that these criteria are not likely to be considered by HTA bodies. In the third scenario, only the criteria of *Manufacturing Complexity and Unique Indication or Not* were excluded from the analysis to establish how inclusion of *Level of Research Undertaken and Level of Effectiveness Uncertainty* might influence the outcomes compared to Scenario 2 (Table 2).

**Table 2:** Scenario testing with weights applied to each criterion

Criteria	Weights applied in each scenario		
	Scenario 1 (Base Case)	Scenario 2	Scenario 3
Rarity	11.1%	14.0%	10.0%
Level of research undertaken	11.1%	0.0%	20.0%
Level of effectiveness uncertainty (robustness of endpoints)	11.1%	0.0%	10.0%
Manufacturing complexity	11.1%	0.0%	0.0%
Follow up measures or Monitoring	11.1%	6.0%	15.0%
Disease severity	11.1%	30.0%	15.0%
Available alternatives (Unmet medical need)	11.1%	20.0%	10.0%
Level of impact on disease (Disease modification)	11.1%	30.0%	20.0%
Unique indication or not	11.1%	0.0%	0.0%

## Results

**Fig. 1:**
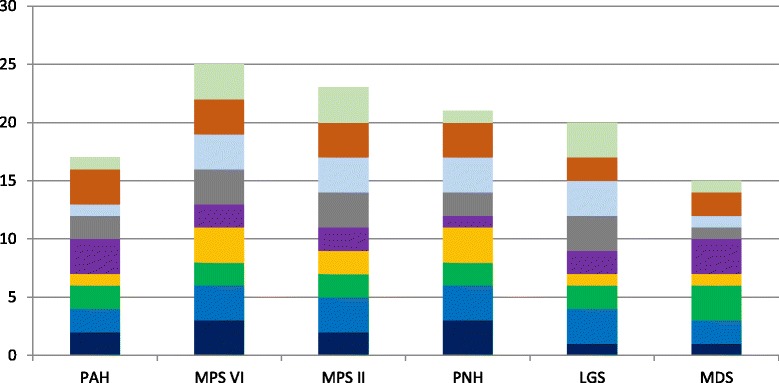
Overall drug scores based on the proposed MCDA framework. PAH: *Pulmonary arterial hypertension*; MPS II: *Mucopolysaccharidosis II*; LGS: *Lennox-Gastaut syndrome*; MPS VI: *Mucopolysaccharidosis VI*; PNH: *Paroxysmal nocturnal haemoglobinuria*; MDS: Myelodysplastic syndromes

**Fig. 2:**
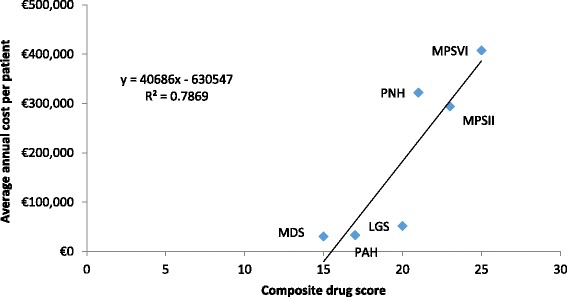
The total drug score in relation to the average annual cost (€) based on UK list prices. PAH: *Pulmonary arterial hypertension*; MPS II: *Mucopolysaccharidosis II*; LGS: *Lennox-Gastaut syndrome*; MPS VI: *Mucopolysaccharidosis VI*; PNH: *Paroxysmal nocturnal haemoglinuria*; MDS: *Myelodysplastic syndromes*

The overall scores achieved for each drug, including the contribution of the individual criteria scores to the total are depicted in Fig. 1. The drug scores were plotted against the average annual cost per patient for each drug as shown in Fig. 2. In the drugs studied, the *R* ^2^ was 0.7869 suggesting a strong correlation between the drug scores and the average annual cost per patient. In other words, the higher the drug’s aggregate score, the more likely it was to have a high average per patient cost.

**Fig. 3:**
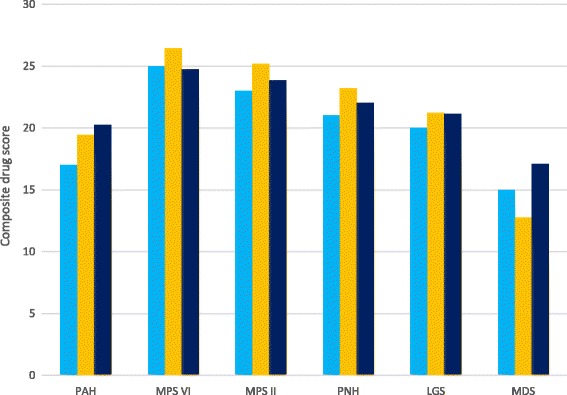
Scenario testing of the criteria with different weighting applied in each scenario. Note: Criteria excluded in Scenario 2: Level of research undertaken; Level of effectiveness uncertainty; Manufacturing complexity; Unique indication or not. Criteria excluded in Scenario 3: Manufacturing complexity; Unique indication or not. PAH: *Pulmonary arterial hypertension*; MPS II: *Mucopolysaccharidosis II*; LGS: *Lennox-Gastaut syndrome*; MPS VI: *Mucopolysaccharidosis VI*; PNH: *Paroxysmal nocturnal haemoglinuria*; MDS: *Myelodysplastic syndromes*

The scenario analyses demonstrate that by applying different weights to the criteria, the ranking of the drugs change, in particular, for those drugs whose average annual cost per patient features on the higher end of the scale (Fig. 3).

## Discussion

This study sought to test the MCDA framework proposed by Hughes-Wilson et al. [8] to establish if such a simple framework, both in the suggested criteria and the simplistic scoring system, could be used to inform in decision making in the reimbursement of drugs. MCDA is a framework that through the choice of appropriate criteria, can assist decision makers in healthcare. We believe that although the suggested framework provided a tool worth testing, and provided initial insights into the application of MCDA as a method of assessing orphan drugs, it did not capture some of the essential criteria. This might be because the proposed framework may have been developed from a pharmaceutical company perspective, since it is unlikely that a HTA body would consider M*anufacturing complexity* and the *Unique indication or not* for which a drug is licensed as relevant criteria when assessing orphan drugs for reimbursement decisions.

Furthermore, other important limitations of this MCDA framework would need to be addressed before MCDA frameworks could be considered. One such limitation is the scoring system that was proposed and therefore adopted. It is a simplistic numerical scoring system from 1 to 3 that implies that a change in a criterion from a score of 1 carries the same value as the change from a score of 2 to 3. Similarly, the proposed framework did not allow for weighting of the criteria, yet in reality it can be anticipated that criteria should be weighted differently to accommodate the degree of importance of criteria.

A further limitation of the study is that the scenario testing adopted in this study excluded those criteria such as *Level of Research Undertaken*, *Level of Effectiveness Uncertainty, Manufacturing Complexity and Unique Indication or Not*. Whilst this was done on the basis that these criteria are not likely to be taken into account by HTA bodies, it limits the number of criteria that are then reflected fully. Whilst this proposed framework may inform in the decision making for orphan drugs, it does not offer insights for comparing resource allocation of orphan drugs with non-orphan drugs. Future MCDA frameworks should include a broader range of criteria that better reflect the *Efficacy* and *Safety* of the drugs. This may potentially lead to MCDA frameworks being disease-specific so that *Efficacy* can be captured in relation to the disease. The downside of such a framework however, would be that it would not allow for comparisons of drugs across different therapy areas.

One of the criticisms of current HTA cost-effectiveness analyses is that these are largely viewed from a healthcare perspective, and may fail to include the patient and societal perspectives [26]. This also raises questions as to whether MCDA should be applied from a single perspective or whether attributes can reflect varied perspectives. Future MCDA models could potentially include criteria to represent multiple perspectives, that will allow for several additional analyses of the impact of criteria on decision making.

Since resources in healthcare at both the public and privately funded levels are scarce, cost-effectiveness analyses have been adopted in decision making to ensure the efficient use of finite resources with a view to maximising health benefits [27, 28]. This intention of the proposed MCDA framework [8] was to suggest a robust way for decision makers to conduct a comparative assessment of orphan drugs. However, the authors of the proposed MCDA framework did not suggest how the tool could be used to address issues of healthcare resource allocation. We would expect that the use of MCDA will first have to undergo further studies and an adjudication or validation process, and that it is likely that MCDA composite score thresholds of affordability and healthcare resource efficiency might be used.

We had aimed to compare the composite scores for each of the drugs with published ICERs as a way of establishing if parallels could be drawn between the two sets of data. However, due to the paucity of published ICERs for all six drugs in the same country, we were unable to perform this comparison. We suggest that future MCDA frameworks take ICERs into account as an external “validation”.

Increasingly MCDA tools are being developed for healthcare purposes. For example, The Evidence and Value: Impact on Decision Making (EVIDEM) group proposed a “core” MCDA framework for use in healthcare [29]. The aim of the EVIDEM framework is to support the prioritisation of a broad range of healthcare interventions, such that priority is given to the intervention that obtains the highest rank. Because the EVIDEM MCDA model was developed for a wide range of healthcare decisions (including services, products, drugs) it is a complex model with far more criteria than the MCDA framework adopted in this study.

The region of Lombardia in Italy has adopted an MCDA approach to regulate the introduction of new health technologies. Their MCDA is based on the EVIDEM framework. The introduction of this formal MCDA model stemmed from the desire to balance goals of continuous innovation with the needs of steady cost containment, and to instill uniformity and transparency in a process that may be highly complex. While subjectivity cannot be completely removed, the framework seeks to minimise discretion in decision making and to produce decisions perceived as legitimate by all the stakeholders [30]. The EVIDEM framework has also been used in Canada by healthcare payers, in a way of bridging MCDA with health technology assessments [31].

Since many rare diseases can have severe consequences on health and health-related quality of life [32], *Disease Severity* is a criterion of substantial importance in assessing the benefits of orphan drugs on health-related outcomes in patients. Although one would expect *Disease Severity* to influence reimbursement decision-making, its application in reimbursement decisions is not widely known. By contrast, the MCDA tool allows for consideration of the severity of a disease. Because of the often complex nature of rare diseases, we believe that *Disease Severity* should be sub-divided into 4 sub-criteria of *Disease-related mortality, How symptomatic the disease is, Mental status (anxiety / depression), Physical implications and/or disability.*

The criterion *Unmet Need* is one that deserves further clarification for its application in MCDA. Whilst the framework does not allow for it to have sub-criteria, we feel that the unmet need in a disease is not only dependent on the number of other available drugs for the disease, but also the benefit that is likely to be gained from the alternative treatment(s). In the highly specialised technology (HST) guidance in England, NICE does consider the *Unmet need* when assessing drugs for very rare conditions [33]. In The Netherlands, the ministry introduced a temporary scheme for orphan drugs, that recognises both the lack of cost-effectiveness data and the high unmet need that new orphan drugs address [34]. Similarly, the criterion *Level of research undertaken* is simplified, and we believe that it should reflect the three sub-criteria *of Level of trials undertaken, Trial duration, Size of trial.*

A criterion that we would recommend in future MCDA models is *Treatment Convenience.* Our rationale for this suggestion is that since some drugs may need to be administered regularly by non-oral routes i.e injection or intravenously, these may impact significantly on overall treatment costs, an aspect of treatment worth including in drug assessments. Taking account solely of the drug cost is not an accurate reflection of the total cost of a patient’s treatment.

We appreciate that the framework proposed by Hughes-Wilson et al. [8] was intended as a basic starting point for the adoption of MCDAs in assessing orphan drugs. However, its simplicity fails to capture a key criterion that HTA bodies would consider, in particular that of *Safety*. Following further research, we recommend that *Safety* should be divided into 3 sub-criteria that include *Serious adverse events (in clinical trials), Drug discontinuation due to adverse events, and Treatment-related mortality.*

A simple ordinal scoring system was applied in this model, with equal importance between grades. Despite potential criticism about the imprecision of a simple numerical scoring system, the rationale for its use is due to its simplicity and that it does not require an expert panel to adjudicate the value of one criterion against another, as would be the case in outranking methods [35], satisficing methods [36] and value measurement methods [36]. Furthermore, it does not require the use of special computer software.

In this exploration of the proposed MCDA model, we adopted simple scenario testing to measure the impact of weighting the criteria differently. Although the 3 scenarios demonstrated a slight difference in the ranking of the drugs, weighting is none the less a feature of MCDAs that requires further investigation. A recent publication [37] which was identified in the literature review examined eight weighted criteria that were considered important for orphan drug approvals. These were categorised by either the Impact of disease/extend of unmet medical need*,* which included *Availability of existing treatment, Disease survival prognosis with current standard of care (SOC), Disease morbidity and patient clinical disability with current SOC, Social impact of disease on patients’ and carers’ daily lives with current SOC*, or the Impact of new medicine, which considered the criteria of *Treatment innovation, Evidence of treatment efficacy, Evidence of treatment safety, Social impact on treatment on patients’ and carers’ daily lives.* The 8 criteria were weighted in different scenarios by two groups, of which one were “clinical/economic experts” and the other were patient advocates. The clinical/economic experts put more weight on efficacy, whereas the patient advocates weighed treatment efficacy and impact on daily lives equally. While the scenario testing proved insightful, it is worth noting that some of the criteria included in the study would not necessarily be ones that HTA bodies consider, such as those that focus on the social aspects of the rare disease and the social impact of the new drug. Due to the differences in the criteria considered in the aforementioned study [37], we felt that we could not adopt their weighting preferences in testing our model. However, any weighting that might be applied to a MCDA model can be tailored by an HTA body or decision maker to reflect local preferences.

The R-squared value of 0.7869, the response variable variation that is explained by a linear model, suggests a good correlation between the average annual cost per patient and each drug’s aggregate score. This implies that as the MCDA score per drug increases, so does the average annual cost. However, there are limitations to using R-squared in that it cannot determine whether or not the coefficient estimates and predictions are biased. Additional regression analyses should be included in future studies on MCDAs to test the best-fit.

It would be ideal to subject a new method or model for assessing drugs, irrespective of the perspective from which it is conducted, to an external process of validation. To the best of our knowledge, the framework that the authors proposed [8] has not been tested nor validated. At this point, the proposed model is conceptual and would be subjected to validation once the criteria and other aspects, such as the methodology pertaining to the scoring and weighting, have been refined.

A drawback of MCDA frameworks is that they do not inform on the budget impact of the drugs in question. However, it is fair to point out that cost-effective analyses that report on ICERs also do not express the budgetary impact and consequently do not inform on the viability of adopting a new technology from the healthcare system perspective [38, 39]. We suggest that when developing MCDA tools in the future, it might be appropriate to include a criterion to assess the budget impact, based on the likely patient population that would be treated with each drug in the health economy for which the tool is developed. This process could be standardised by using the budget impact template provided by the Scottish Medicines Consortium [40].

In the future, a key criterion that should be included in MCDAs is the health-related quality of life (HRQoL), which is frequently reported on in clinical trials, and which is likely to reflect the impact of the disease and new treatment on the patients than the social aspects captured by Sussex et al. [37].

## Conclusion

The framework proposed by Hughes-Wilson et al. [8] has provided a small insight into the application of MCDAs to orphan drugs. At the time of this study, the ISPOR MCDA Task Force reports and guidelines had not been published, and in future these should be closely considered. It is worth noting that several other criteria would offer additional insights to the overall product aggregate. In a market constrained by costs, a product aggregate score that includes criteria for innovation and HRQoL could assist decision makers considerably. The strength of MCDAs in reimbursement decisions for orphan drugs is that they provide transparency and robustness, and unlike traditional HTA methods, assess more than merely cost-effectiveness. Defining the criteria at the outset is crucial to ensure that overlap between criteria is avoided. Furthermore, it is essential that the criteria are not selected merely to favour a preferred outcome. Weighting the criteria may be complicated, and dependent on the perspective of the assessment [41]. Future work will include research to understand the weights of different criteria and how they affect the outcomes; and to compare HTA decisions with MCDA outcomes.

### Literature search terminology

“orphan drug”, “multi-criteria decision analysis”, “MCDA”, “MCDA + healthcare”, “multi-criteria decision analysis + healthcare”, “multi-criteria decision analysis + orphan drug”

## Abbreviations

BNF: British National Formulary
EPAR: European public assessment reports
HRQoL: Health-related quality of life
HST: Highly specialised technology
HTA: Health technology assessment
ICER: Incremental cost-effectiveness ratio
LGS: Lennox-Gastaut syndrome
MCDA: Multi-criteria decision analysis
MDS: Myelodysplastic syndromes
MPS II: Mucopolysaccharidosis II
MPS VI: Mucopolysaccharidosis VI
NHS: National Health Service
NICE: National Institute for Health and Care Excellence
PAH: Pulmonary arterial hypertension
PNH: Paroxysmal nocturnal haemoglobinuria
SMC: Scottish Medicines Consortium
SPC: Summary of Product Characteristics
